# Ephedra Vulgaris

**Published:** 1892-09-10

**Authors:** 


### THERAPEUTICS.

### EPHEDRA VULGARIS.

Ephedra vulgaris has been used by Dr. Becktin,* of St. Petersburg. It has long been employed by the Russian peasantry as a domestic remedy in rheumatic affections, and likewise in syphilis. The author uses a decoction of sixty grains of his powdered roots and twigs in about seven ounces of water, the dose of which is about a tablespoonful every two hours. He has found it most useful in acute articular and muscular rheumatism. In about forty-eight hours its administration is followed by considerable relief from pain with diminution of the febrile symptoms. In about eight or ten days the patient may be said to be cured. Even in those cases where pericarditis existed as a complication, this also is stated to have disappeared with the other rheumatic manifestations. In chronic rheumatic affections the author states that the therapeutic action of ephedra is less favourable and more slowly produced; though here also he has obtained marked improvement. The remedy possesses laxative, diuretic, and diaphoretic properties. Professor Nagois, of Tokio, has isolated the alkaloid ephedrine from ephedra vulgaris. This, injected into dogs and cats, produces general convulsions, mydriasis, and exophthalmia. A different species of the wild raspberry, rubus chamæmorus, the yellow raspberry, has also been utilised in the form of a decoction as a diuretic drug. Professor Popow extracted an acid in the form of a colourless powder from the extract of the flowers. With bases it forms crystalline salts, which are very soluble in water. The acid is very readily dissolved by alcohol, but is only sparingly soluble in water, and it is regarded by the Professor as the active principle of the plants.

* *New York Medical Journal*, November 28th, 1891.

This substance produces its diuretic effect without stimulating the heart's action or increasing arterial tension. Bouchenoff has used an infusion made from sixteen parts of the leaves to 180 parts of boiling water. He administered it as a diuretic in doses of five or six drachms daily, in cases of nephritis, cirrhosis of the liver, cardiac affections, chloroanæmia, and in one case only did it fail to produce a diuretic effect.

The conclusions arrived at by Becktin may be summarised * (1) The steppe raspberry is decidedly one of the most valuable anti-rheumatic remedies yet known. (2) The best results are obtained in cases of acute articular and muscular rheumatism. Even on the second day of the treatment pain greatly decreases, and the pulse and respiration become slower while growing fuller and stronger in a couple of days, the temperature returns permanently to normal, and articular swelling completely disappears, and in seven or ten days the patient is quite well. In the complicated case the pericarditis subsided, together with the other morbid pnenomena; (3) In chronic cases the remedy acts somewhat less strikingly and less rapidly. Of the eight cases, in four (two muscular, two articular) a great improvement was obtained. (4) In rheumatic osteo-myelitis and sciatica, however, the remedy seems to produce only slight and temporary amelioration. It may be added that in both the cases all ordinary anti-rheumatic drugs (salicylate of sodium, antipyrin, antifebrin, salol, phenacetin, &c.) utterly failed to afford any relief. (5) Ephedra invariably has a beneficial effect in the gastro-intestinal tract, habitual constipation disappearing, &c. (6) The remedy also appears to possess a diuretic action, which is especially pronounced in cases of articular rheumatism.

The place of ephedra vulgaris in the treatment of rheumatism may be compared to that of actea racemosa, to the nature and properties of which we alluded in a recent issue.

* *British Medical Journal*, August 8th, 1891.

